# Benzylaminopurine and Abscisic Acid Mitigates Cadmium and Copper Toxicity by Boosting Plant Growth, Antioxidant Capacity, Reducing Metal Accumulation and Translocation in Bamboo [*Pleioblastus pygmaeus* (Miq.)] Plants

**DOI:** 10.3390/antiox11122328

**Published:** 2022-11-24

**Authors:** Abolghassem Emamverdian, Yulong Ding, Mohammed Nasser Alyemeni, James Barker, Guohua Liu, Yang Li, Farzad Mokhberdoran, Parvaiz Ahmad

**Affiliations:** 1 Co-Innovation Center for Sustainable Forestry in Southern China, Nanjing Forestry University, Nanjing 210037, China; 2 Bamboo Research Institute, Nanjing Forestry University, Nanjing 210037, China; 3 Botany and Microbiology Department, College of Science, King Saud University, Riyadh 11451, Saudi Arabia; 4 School of Life Sciences, Pharmacy and Chemistry, Kingston University, Kingston-upon-Thames KT1 2EE, UK; 5 Department of Mathematical Sciences, Florida Atlantic University, Boca Raton, FL 33431, USA; 6 Department of Botany, Govt Degree College, Pulwama 192301, Jammu and Kashmir, India

**Keywords:** metal toxicity, oxidative stress, reactive oxygen species (ROS), bamboo, antioxidants, plant stress tolerance, translocation factor, bioaccumulation factor, phytoremediation

## Abstract

An in vitro experiment was conducted to determine the influence of phytohormones on the enhancement of bamboo resistance to heavy metal exposure (Cd and Cu). To this end, one-year-old bamboo plants (*Pleioblastus pygmaeus* (Miq.) Nakai.) contaminated by 100 µM Cd and 100 µM Cu both individually and in combination were treated with 10 µM, 6-benzylaminopurine and 10 µM abscisic acid. The results revealed that while 100 µM Cd and 100 µM Cu accelerated plant cell death and decreased plant growth and development, 10 µM 6-benzylaminopurine and 10 µM abscisic acid, both individually and in combination, increased plant growth by boosting antioxidant activities, non-antioxidants indices, tyrosine ammonia-lyase activity (TAL), as well as phenylalanine ammonia-lyase activity (PAL). Moreover, this combination enhanced protein thiol, total thiol, non-protein, glycine betaine (GB), the content of proline (Pro), glutathione (GSH), photosynthetic pigments (Chlorophyll and Carotenoids), fluorescence parameters, dry weight in shoot and root, as well as length of the shoot. It was then concluded that 6-benzyl amino purine and abscisic acid, both individually and in combination, enhanced plant tolerance under Cd and Cu through several key mechanisms, including increased antioxidant activity, improved photosynthesis properties, and decreased metals accumulation and metal translocation from root to shoot.

## 1. Introduction

In recent years, due to growing urbanization and industrialization, the accumulation of excess heavy metal has become a major threat to human health, polluting farmland and forestland everywhere [1]. Among them, Cd and Cu are the two most prevalent heavy metals in Chinese forestland [2]. There is substantial evidence that Cd is phytotoxic to plant growth [3]. Cadmium causes plants to reduce transpiration by closing the stomata. [4]. However, cadmium toxicity alters carbohydrate and nitrogen metabolism [5] and changes the functioning of the membranes by triggering lipid peroxidation [6]. In addition, it causes plant oxidative stress as evidenced by increasing H_2_O_2_ content [7]. Cu is recognized as a pivotal micronutrient for plant growth. However, high concentrations of Cu are harmful to plants and can cause necrosis and chlorosis, leaf discoloration, and stunting, as well as restricting plant root growth [8]. Additionally, an excess of Cu results in the overproduction of reactive oxygen species (ROS), which can trigger lipid peroxidation in the cell membrane and osmotic stress in plants [9,10]. Furthermore, it can disrupt the primary process of plant photosynthesis [11,12].

Among plant hormones, abscisic acid (ABA) and 6-benzylaminopurine (6-BAP) are the two growth regulatory phytohormones that play a pivotal role in the regulation of plant response to abiotic stress by influencing physiological, morphological, and biological processes in higher plant species [13,14]. ABA, also recognized as a stress hormone, has a signaling role in encoding the genes associated with antioxidant enzyme responses under stress [15]. This could improve the plant defense system against abiotic stresses, such as metal ions [16], salinity [17], and drought [18]. On the other hand, root meristems are one of the primary sources of ABA production in plant organs, which can serve as an initial protection against heavy metal toxicity in the rhizosphere. ABA’s interaction with metal ion reduces the ions’ activity and limits metal translocation from the root to aerial plant parts [19]. In a study on *Phragmites* and *Typha* exposed to Cd toxicity, the accumulation of ABA in the roots was significantly greater than in the shoot, resulting in an increase in plant tolerance to cadmium toxicity [19]. 6-BAP is a major growth phytohormone and is classified as a cytokine, which can boost plant tolerance to abiotic stress by scavenging ROS and free radicals [20]. This functionality has been demonstrated in numerous studies on perennial ryegrass (*Lolium perenne* L. cv. Pinnacle) under salinity stress [21], cucumber (*Cucumis sativus* L.) under light stress [22], mustard (*B. juncea*), and switchgrass (*Panicum virgatum* L.) under heavy metal stress [13,23]. The mechanism by which 6-BAP reduces the toxicity of metal ions in plants is achieved through exerting a positive effect on photosynthesis by reducing the degradation of chlorophyll, accelerating protein biosynthesis to form chloroplasts, accumulating different amino acids, controlling and regulating the metabolism of N and C, with the most important mechanism being the stimulation of antioxidant enzyme activity to scavenge ROS components (H_2_O_2_, O_2_^•−^). Additionally, it includes reduction of malondialdehyde (MDA) content by reducing plant lipoperoxidation [22,24,25]. On the other hand, it has been reported that there is a positive relationship between 6-BAP and ABA levels, which means that the presence of 6-BAP can increase the accumulation of ABA in plant parts, especially in the root meristem, which eventually improves plant tolerance to metal toxicity through increasing antioxidant activity, regulating stomata aperture and enhancing the photosynthesis process, as well as enhancing apical dominance in stems and plant growth [19]. Additionally, we hypothesized that 6-BAP and ABA in isolation can increase the metal resistance of bamboo plants. However, the combination of 6-BAP and ABA could be more effective by boosting the accumulation of ABA induced by 6-BAP, which can lower the translocation and accumulation of metal toxicity from the root to the aerial plant organs (stem and shoot).

Bamboo (*Bambusoideae*) species are known for their rapid growth, high biomass, and high yield production. In a short period of time (a few years), they produce an important non-timber forest product (NTFP) and cover a large portion of tropical and subtropical forestland [26]. Additionally, they encompass a vast portion of the global forestland [27]. Approximately six million hectares of China’s forests are allocated to bamboo species, which are categorized into 500 species in 48 genera [28]. On the other hand, bamboo is one of the main economic and subsistence sources for local people in the southwestern and southern regions of China [29]. Asians and people from all around the world consume bamboo shoots for their high fiber and low fat content [30]. Due to its high biomass yields and high capacity for metal ion extraction through its roots, bamboo plants are a viable alternative for phytoremediation and the elimination of environmental contamination [31]. On the other hand, certain bamboo species are used in commercial projects (gardening and beautification) as aesthetic plants, and their presence can be a useful indication of plant tolerance to metal stress from sewage and air in urban environments. Consequently, bamboo can be used to clean the air and eliminate sewage pollution in urban areas [32]. Regarding this issue, we selected the *Pleioblastus pygmaeus* bamboo plant, which is an evergreen species of bamboo plant with a 30–50 cm height that can grow in all types of soil (acidic, basic (alkaline), and neutral) and is suitable for landscaping purposes [33,34,35]. It is utilized as an ornamental and landscaping plant in Jiangsu province. In recent decades, heavy metals, particularly cadmium and copper, have become a major concern for human health and environmental safety in China and across the world, posing a direct hazard to water supplies and agricultural and forestland soils [36]. Therefore, excess heavy metals due to human activities imperil the health and economy of the local people of this region whose livelihood is dependent on the bamboo plants. Since there is a lack of sufficient study in this area on bamboo species, further research is required to identify novel bio-applications for alleviating plant and environmental toxicity. The goal of this study is to evaluate the positive effect of two phytohormones in their individual and combined forms on enhancing bamboo plant tolerance to two heavy metals (Cd and Cu), with an emphasis on the mechanism involved. To the best of the authors’ knowledge, this is the first study on the combined use of two phytohormones for the reduction of Cd and Cu toxicity and the enhancement of bamboo species’ tolerance under stress conditions, which could aid in plant and environmental detoxification.

## 2. Materials and Methods

### 2.1. Plant Material and Growth Conditions

**Figure 1:**
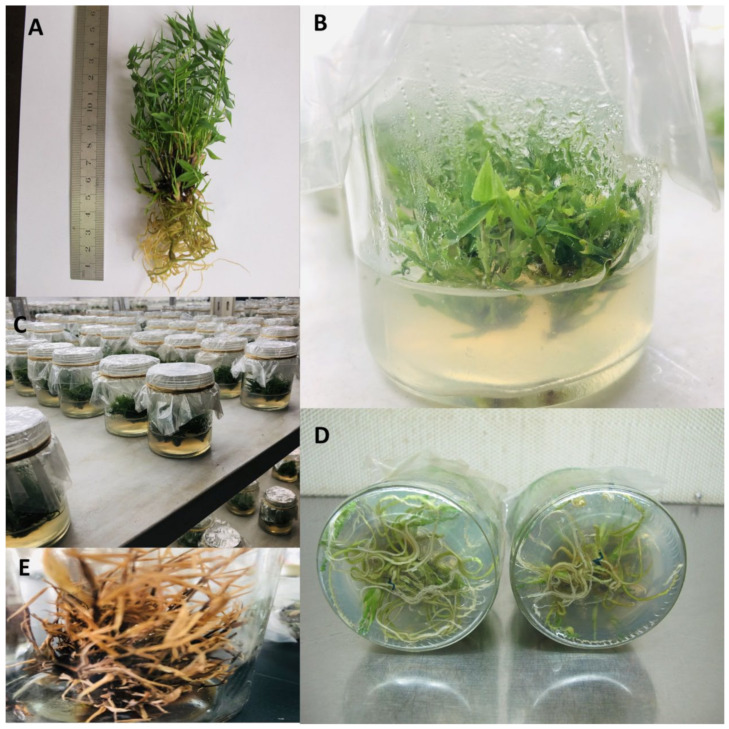
Bamboo plant phenotypes in vitro condition. (**A**) Bamboo height in the experiment (**B**) Bamboo treatments in the glass petri dish with culture medium (**C**) Bamboo treatments in the controlled plant tissue culture chamber (**D**) Roots proliferated from the immature shoots in bamboo species (*Pleioblastus pygmaeus*), (**E**) Exposure of the bamboo plant to 100 µM cadmium.

One-year-old branches of *Pleioblastus pygmaeus* from a single clone were utilized as plant treatments. Since 1982, *Pleioblastus pygmaeus* has been one of the oldest species at the Bamboo Garden of Nanjing Forestry University in Nanjing, Jiangsu, China. In the pre-treatment stage, 10 mm long nodal explants were cultured in a MS medium [37,38]. Hence, the bamboo shoots were initially formed and grown in the MS medium containing 0.6 mL kinetin (KT), 8–12 g L^−1^ agar, and 28 g L^−1^ sucrose. In the next step, to proliferate roots from immature shoots, 1 L of MS medium containing 0.6 mM myoinositol and 4 µM nicotinic acid, 1.2 µM thiamine-HCl with 4 µM pyridoxine, and 1 mg L^−1^ Indole-3-acetic acid (IAA) was provided and then 28 g of sucrose with different experimental treatments, including 100 µM Cd, 100 µM Cu with 10 µM 6-benzyl amino purine, and 10 µM abscisic acid in both single and combined forms were added to the solution before the pH was adjusted to 5.8 ± 0.1. Next, 8–12 g L^−8^ agar was mixed in. The MS medium was transferred to a microwave oven (China Energy label) and subsequently heated for 30 min at 120 °C. Afterwards, it was stored in glass petri dishes (60 mm diameter and 90 mm height) with 100 mL culture medium. Then, the MS medium was sterilized in a HiClave HVE-50 autoclave. The breeding room was sterilized with UV radiation (10–380 nm) in this study. Bamboo plantation was then carried out in a single ultraviolet-sterilizer incubation hood (Air Tech, Rutherford, NJ, USA) with white fluorescent lights at temperatures of 17–23 °C in dark periods and 24–28 °C in light periods. The pre-incubated plant species treatments were then transferred to a controlled plant tissue culture chamber and maintained there with a white fluorescent light source in the incubator, with 16 h of photoperiod and temperatures of 17/22 °C and 30/25 °C in the dark and light periods, respectively (Table 1, Figure 1). This investigation was conducted over the course of three weeks.

6-benzyl amino purine, abscisic acid, cadmium, and copper were purchased from Nanjing Jiancheng Company. In this study, the concentrations of Cd and Cu, as well as those of phytohormones, were determined based on the various ranges of bamboo resistance found in our previous research [39].

In the final step, once the growth process was complete, the treated bamboos were removed from the MS medium cleaned and washed.

### 2.2. Measurement of Plant Development Related Parameters

Different bamboo samples were divided into root and shoot samples, which were used throughout the final stage of the research project. Fresh shoots were preserved at −80 °C in tiny fractions for biochemical analysis after being placed in a cold environment. The remaining root and shoot sections were used to determine shoot and root fresh weight (FW) and then transferred to a dry oven at the optimal temperature, i.e., 85 °C, for 48 h. The shoot dry weight (SHDW) and root dry weight (RDW) were recorded. In this study, the shoot length was measured twice, at the beginning and end of the experiment. Then, the difference between the two measurements was determined as shoot length.

### 2.3. Determination of Photosynthetic Parameters

Chlorophyll pigments, including chlorophyll a, chlorophyll b, carotenoid content, and total chlorophyll, were measured using the Lichtenthaler method [40]. In this study, 0.5 g of bamboo leaf samples were pulverized in a mortar using liquid nitrogen (LN) for their determination. The powder was then added to 20 mL of 80% acetone at an optimal temperature between 0 and 6 °C. The extracted solution was then centrifuged at 8000× *g* for 12 min. Finally, a UV/vis spectrometer was used to determine chlorophyll a, chlorophyll b, and carotenoid content based on their absorbance at 663, 645, and 470 nm respectively. The following formulae were used to calculate mg/g of fresh weight values:

Chlorophyll a value = 12.25 × (A value of 663 nm) − 2.79 × (A value of 647 nm).(1)

Chlorophyll b value = 21.50 × (A value of 647 nm) − 5.10 × (A value of 663 nm).(2)

Carotenoid content = 1000 × (A value of 470 nm) − 1.82 × (Chl a) − 95.15 × (Chl b)/225.(3)

Total − Chl = Chl a + Chl b(4)

To determine the fluorescence characteristics, a chlorophyll fluorescence imager machine (CFI) (England) was utilized for 35 min in a dark-adapted environment. In this process, the light fluorescence index was measured at 700 µmol m^−2^ s ^−1^, carried out in an illumination incubator used to activate actinic light. The major fluorescence indices were: (A) actual photochemical efficiency in PSll (ɸPSll); (B) photochemical quenching coefficient (qP); (C) effective photochemical efficiency in PSll (Fv′/Fm′); (D) maximum photochemical efficiency in PSll (Fv/Fm); and (E) non-photochemical quenching (NPQ).

### 2.4. Measurement of Biochemical Parameters

#### 2.4.1. Oxidative Stress Indicators

Hydrogen peroxide (H_2_O_2_) was measured according to the protocol proposed by Junglee et al. [41]. In accordance with this, the leaf samples were mixed with 0.2% trichloroacetic acid, followed by 100 mM potassium phosphate as a buffer with pH = 7.1 and 1 M of potassium iodide. The absorbance of H_2_O_2_ at the final step was measured at 390 nm. The superoxide radical (O_2_^•−^) content was measured using the Li method [42], achieved through absorbance at 530 nm and 25 °C. A standard curve and a nitrogen dioxide radical (NO_2_) were employed for measuring O_2_^•−^. The soluble protein was measured using the Bradford method [43], obtained by combination of 0.1 Coomassie Brilliant Blue (G25), 50 mL 90% ethanol, 1000 mL water, and 100 mL H_3_PO_4_. Then, the alternation of the protein levels was recorded by one spectrometer, which showed soluble protein content. In this study, thiobarbituric acid reactive substances (TBARS) were utilized as an indication of lipid and cell peroxidations, according to De Leon and Borges [44]. Additionally, it was measured by an absorbance reading at 532 nm with TBARS (represented in µM g^−1^ leaf FW). The activity of lipoxygenase (LOX) was measured according to the protocol of Grossman and Zakut (1979) [45], and Sekha and Reddy (1982) [46]. For this purpose, 10 μL linoleic acid was mixed with 25 mL of the 0.1 M sodium tetraborate containing 0.1% Tween 20. In the next step, 2.9 mL of 0.1 M phosphate buffer (pH 4–5) was added to 0.1 mL of the obtained substrate and then measured in a UV spectrophotometer device (Beijing Purkinje TU-1810UV-vis spectrometer, Beijing, China) with enhancement in absorbance at 234 nm measured every 30 s. Electrolyte leakage (EL) was recorded in accordance with the protocol proposed by Senthilkumar, et al. [47]. In this experiment, 20 mL of the deionized water was mixed with 0.4 g of the leaf sample. Having the resultant mixture maintained at 30 °C for 4 h, the electrical conductivity was estimated with the notion of EC_1_. The treatments were then kept in an autoclave at 110 °C for 20 min, after which the electrical conductivity was measured with the notion of EC_2_. The total EC was then calculated according to the formula shown below.

EC (%) = EC_1_/EC_2_ × 100 (5)

#### 2.4.2. Antioxidant Enzyme Activities

Approximately 0.5 g of the bamboo leaves were shredded and ground in a mortar and pestle, and the resulting powder was mixed with liquid nitrogen (LN). Then, it was kept at 2–8 °C. Next, 3 mg of phosphate buffer with a pH of 7.6 was mixed with the powder produced in a test tube. It was then centrifuged at 2500–3500× *g* at 5 °C for 15 min. Therefore, the eventual supernatant was utilized to determine the antioxidant activity.

The superoxide dismutase (SOD) activity (E.C. 1.15.1.1) was estimated using nitro blue tetrazolium (NBT) photoreduction and absorbance at 560 nm in a single UV spectrophotometer device (Beijing Purkinje TU-1810UV-vis spectrometer) according to Senthilkumar method [48] which was determined using the standard assay procedure. The peroxidase activity (POD, E.C. 1.11.1.7) was measured based on the Liu method [49]. In this method, 2.99 mL of sodium phosphate buffer (50 mM, pH 6.0) containing 4.4 mM H_2_O_2_ and 18.2 mM guaiacol was added to 10 µL volume of enzyme solution as substrate. POD was determined at an absorbance of 470 nm (0.001 per minute and 25 °C). Catalase (CAT) activity (E.C. 1.11.1.6) was estimated by using the H_2_O_2_ extinction coefficient of 39.4 M^−1^ cm^−1^ and lowering the absorbance to 240 nm [50]. In this test, the mixture for the reaction purpose included 10.5 mM H_2_O_2_ and 50 mM potassium phosphate buffer (pH 7.0) which ran at 25 °C for 2 min. The activity of ascorbate peroxidase (APX) (E.C. 1.11.1.11) was measured utilizing the Nakano and Asada protocol [51] in the decrease of absorbance at 290 nm with an absorbance coefficient of 2.8 mM^−1^ cm^−1^. For this purpose, the reaction mixture contained 0.2 mM EDTA, 50 mM potassium phosphate buffer (pH = 7.0), 0.25 mM H_2_O_2,_ and 0.5 mM ascorbic acid at 25 °C with the addition of enzyme extract and H_2_O_2_. Glutathione reductase (GR) activity (E.C. 1.6.4.2) was measured using modifications to the protocol by [52]. For this index, 100 mM phosphate buffer with (pH = 7.8) was added to the extract samples. The present mixture was added to 0.05 mM nicotinamide adenine dinucleotidephosphate (NADPH), 3.0 mM oxidized glutathione, 50 μL of the enzyme extract, 0.1 μM EDTA, and 1.0 mL NADPH oxidation. Then, absorbance of the 340 nm in duplicate RG was obtained by the extinction coefficient of the NADPH molar (6.22 mM cm ^−1^). The data expressed as U mg^−1^ protein.

#### 2.4.3. Assessment of Non-Enzyme Antioxidant Activities Including Total Phenolic, Tocopherols, and Flavonols

A mixture of 7 mL of 80% methanol and 0.7 g of the dry leaf samples was prepared and centrifuged at 6500× *g* for 20 min. As a final outcome, a methanolic extract was prepared and utilized in this experiment. The method of Akkol et al. [53] was utilized to obtain the total phenolic. In this experiment, 0.15 mL of methanolic extract was added to 2 mL of 10% Folin–Ciocalteu reagent. Subsequently, the mixture was homogenized by sodium bicarbonate (7%). The absorbance at 765 nm was then measured to determine the total phenolic content. The gallic acid calibration formula (mg GAE g^−1^ plant material) was used to express the total phenolic content. The flavonol content was determined using the Akkol method [53]. In this experiment, methanolic extract (0.4 mL) was mixed with 0.4 mL and 1.5 mL of aluminum chloride (2%) and sodium acetate (5%), respectively. The supernatant was stored for 3 h. Under ambient conditions, absorbance at 445 nm yielded results for flavonol content determination. The routine calibration was applied to the calculation, and the final values were reported by the unit of mg RE/g of plant material. The tocopherol content was determined using the protocol developed by Li, et al. [54]. In this experiment, 3 mL of ethanol was applied to 0.1 g of the leaf samples. The samples were then centrifuged at 10,000× *g* for 10 min. In continuation of the experimental process, 0.2 mL of bathophenanthroline (0.2%), 1 mM of phosphoric acid, and 0.001 M of ferric chloride were homogenized with 0.1 ML of ethanol extract. The tocopherol concentration was determined by measuring the absorbance at 534 nm. Tocopherol acetate standard (TAC) was utilized as the standard calibration curve, which was represented in terms of mM tocopherol acetate equivalent/g plant material.

#### 2.4.4. Determination of Phenylalanine Ammonia-Lyase and Tyrosine Ammonia-Lyase Activities

The tyrosine ammonia-lyase (TAL) activity was measured using the Berner method [53]. In this experiment, 20 μL of the extracted sample was mixed with 500 μL of boric acid buffer at pH 8.2 and 25 mM tyrosine. The absorbance was then measured at 310 nm for 25 min. The p-Coumaric acid was utilized in this experiment as the standard curve. The activity of phenylalanine ammonia-lyase (PAL) was assessed based on the Berner method [55]. According to the recommendations of this method, 20 μL of extracted samples were added to 500 μL of boric acid buffer with a pH of 7.9 and the absorbance was measured at 290 nm for 25 min. For the standard curve, various concentrations of cinnamic acid were used.

#### 2.4.5. Determination of Total Thiol, Protein Thiol, and Non-Protein

Total reduced thiol content of the samples, total thiol status, protein thiol oxidation, and non-protein thiol states were measured. The samples were homogenized in an ice bath with 0.02 M EDTA, and then 0.5 mL of the bamboo species samples were hemolyzed in 0.3 mL of 0.01 M DTNB and 1.7 mL of 0.2 M Tris buffer with pH = 8.3. The 7.9 mL of absolute methanol was homogenized in the solution’s content, which then reached 10 mL. The obtained supernatant was determined by the amount of 412 nm absorbance, recorded after 12 min. The index of total thiol (TTs) was determined by the extinction coefficient of 13.1 mM^−1^ cm^−1^. To measure non-protein thiols (NPTs), 5 mL of homogenates were mixed with 1.5 mL of 50% TCA and 3.5 mL of distilled water and kept for 10 min. In the subsequent step, 1.8 mL of the supernatant was mixed with 0.2 mL of DTNB and 5 mL of 0.4 M Tris as a buffer with pH = 8.8. Non-protein thiols (NPTs) were obtained by monitoring absorbance at 412 nm for 7 min. In this experiment, protein thiol (PT) content was determined by subtracting total thiol (TT) content from NPTs [56].

#### 2.4.6. Determination of Proline (Pro), Glutathione (GSH), and Glycine Betaine (GB) Content

The content of proline was measured using the Ábrahám method [57]. In this test, 330 mg of the leaf samples were digested with sulfosalicylic acid 3%. The obtained supernatant reacted with ninhydrin. The absorbance of the supernatant was recorded at 520 nm. Free proline levels were calculated using a calibration standard curve. The content of glycine betaine (GB) was determined using the protocol proposed by Grieve and Grattan [58]. In this method, dry samples were mixed in hot water at a temperature of 72 °C. The extract was supplied with potassium tri-iodide solution and 2 N HCl and then mixed and cooled on ice for 2 h. The obtained mixture was added to water and cold 1,2-dichloromethane where two layers were formed. The GB was determined by considering a standard curve of glycine betaine (GB) and the absorbance at 365 nm. The glutathione (GSH) content was estimated by the protocol proposed by Sahoo et al. [59]. Therefore, the bamboo sample (0.1 g) was digested in a solution containing 3 mL of 0.4 mM EDTA and 3% TCA at 5 °C, and subsequently centrifuged at 14,000× *g* for 17 min. Afterwards, 0.2 mM of 5,5-dithiol-bis (2-nitrobenzoic) (DTNB) was added to 0.18 mL of obtained supernatant, along with 2 mL of 50 mM potassium phosphate buffer with pH = 6.9. The result was preserved in a controlled condition at 28 °C for 3 min. Finally, the glutathione (GSH) content was measured by recording the absorbance at 412 nm.

### 2.5. Determination of Cadmium and Copper Accumulation in Leaves, Stems, and Roots of Bamboo Species (ICP Analysis)

In this experiment, the samples were prepared based on the Karimi method [60], with certain procedural modifications. To this end, various plant parts, including roots, stems, and leaves, were cleaned and then thoroughly dried in an oven at 120 °C for 4–7 h. In the next step, the samples were mixed with 70% nitric acid at 80 °C for 25 min. The resultant mixture was then centrifuged at 10,000× *g* for 8 min. An atomic absorption spectrometry (AAS) with a Zeeman-effect background correction system and a graphite furnace was used to detect cadmium and copper content in leaves, stems, and roots (Analyst 800, Perkin Elmer, Waltham, MA, USA). This equipment was then employed for the analysis of accumulation of metal ions. Nitric acid (2.5%) was used as a reference for metal ion concentrations by applying a spectral scan. One inorganic target analyst list (TAL) was conducted for standardization of all components at optimal intervals using an unattended automatic analysis run mode.

### 2.6. Determination of the Bioaccumulation Factor (BAF), the Translocation Factor (TF), and the Tolerance Index (TI)

The bioaccumulation factor (BAF) and the translocation factor (TF) were calculated in order to assess the efficacy of varying levels of phytohormones in mitigating the adverse effects of heavy metals. In addition to determining the plant’s resistance to metal toxicity by the addition of phytohormones, the tolerance index (TI) was computed. This was performed using the Souri method [61] and determined using the formula shown below.

Translocation factor (TF) = cadmium and copper content in the plant leaves (µg g^−1^)/cadmium and copper content in the plant roots (µg g^−1^) (6)

Shoot tolerance index (TI) = shoot dry weight (DW) obtained following the application of cadmium and copper treatments (g)/shoot dry weight (DW) obtained by the control treatments (g).(7)

Root tolerance index (TI) = root dry weight (DW) obtained following the application of cadmium and copper treatments (g)/root dry weight (DW) obtained by the control treatments (g). (8)

BAF (roots/stem/leaves) = cadmium and copper accumulation in the root or stem or leaves/cadmium and copper content in the medium.(9)

### 2.7. Statistical Analysis

The current research was conducted using a completely randomized design (CRD), and data analysis was performed using a 2-way factorial design with four repetitions. The R statistical software package was used for the analysis of variance (ANOVA). A Tukey’s test at a confidence level of *p* < 0.05 was used to determine the mean differences between the experimental treatments.

## 3. Results

### 3.1. The Effect of 6-Benzyl Amino Purine and Abscisic Acid on the Plant Development Related Parameters in the Bamboo Species Exposed to Cadmium and Copper Toxicity

According to the results, there was a significant difference between various types of phytohormones and Cd and Cu in the fresh weight of root/shoot, dry weight of root/shoot and shoot length (*p* < 0.001) (Table 2). As the results indicated, while the 100 µM Cd and 100 µM Cu significantly reduced shoot and root dry weight, shoot and root fresh weight, the rate of fresh weight/dry weight as well as shoot length, the addition of 10 µM 6-BAP significantly increased plant biomass and plant growth indices in the bamboo species exposed to Cd and Cu. A similar result was obtained by addition of 10 µM ABA in the bamboo species under 100 µM Cd and 100 µM Cu. However, the greatest increase in shoot and root fresh/dry weight as well as shoot length was associated with the co-application of 6-BAP + ABA in a combination of 100 µM Cd and 100 µM Cu, with a 62% and 60% increase in shoot fresh weight, a 56% and 59% increase in shoot dry weight, a 8% and 8% increase in fresh weight of root, a 43% and 36% increase in dry weight of root and, a 28% and 23% increase in shoot length when compared to their control treatments, respectively. In the case of the rate of root FW/DW, we could not find a significant difference between the control and 10 µM ABA and 10 µM 6-BAP + 10 µM ABA, but, they showed a significant difference in combination forms (phytohormones + heavy metals). Regarding shoot FW/DW, the result showed that there was not any significant difference between the treatments of control, 100 µM Cd, 100 µM Cu, and 10 µM 6-BAP + 10 µM ABA + 100 µM Cu. However, our results indicated that significant differences existed between various phytohormones and Cd and Cu for the rate of FW/DW of the shoot (*p* < 0.001) (Table 2). We suggested that both types of phytohormones (6-BAP and ABA) can increase plant growth, demonstrating a positive impact on bamboo plant growth under heavy metals. Only the combination of 6-BAP and ABA could increase bamboo plant development parameters under Cu toxicity compared to the control treatment. On the other hand, our results showed that phytohormones (6-BAP and ABA) failed to increase the plant growth indexes under Cd toxicity in comparison to the control treatment, but they showed a significant enhancement in the plant growth indices, which was related to the combination of 6-BAP and ABA where they were found to be the most effective at lowering metal toxicity in the plants and improving the plant growth (Table 3).

### 3.2. The Effect of 6-Benzyl Amino Purine and Abscisic Acid on Photosynthetic Parameters Including Chlorophyll Pigments and Chlorophyll Fluorescence

**Figure 2:**
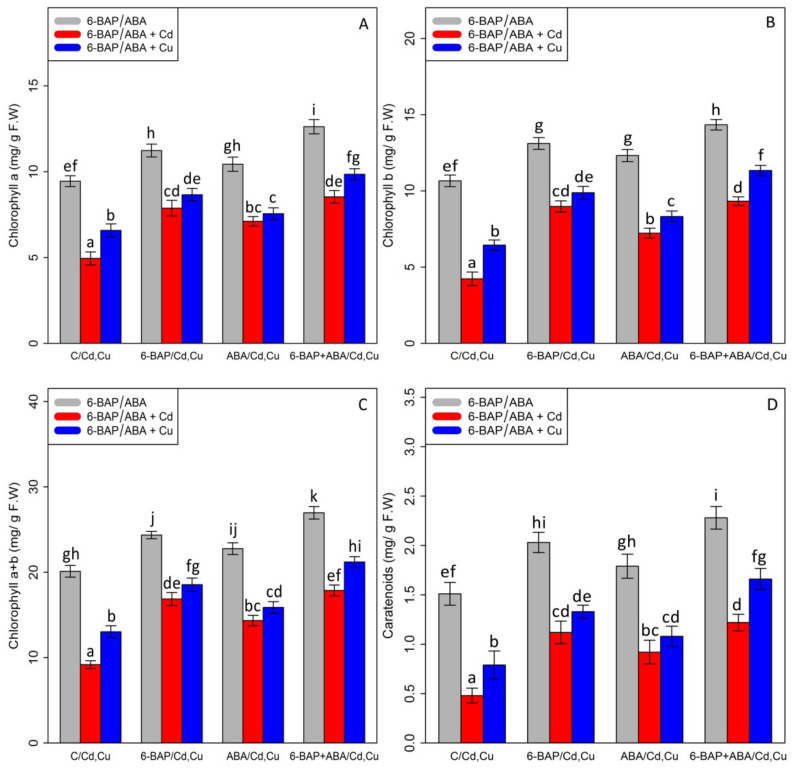
The impact of 6-benzylaminopurine and abscisic acid, alone and in combination, on chlorophyll a (**A**), chlorophyll b (**B**), total chlorophyll content (**C**), and carotenoids (**D**) in the one-year-old *Pleioblastus pygmaeus* exposed to 100 µM Cd and 100 µM Cu. In this figure, a, b, c. etc. letters revealed significant differences between 6-BAP, ABA and 6-BAP + ABA in single and in the combined form with 100 µM cadmium and 100 µM copper according to Tukey’s test (*p* < 0.05).

**Figure 3:**
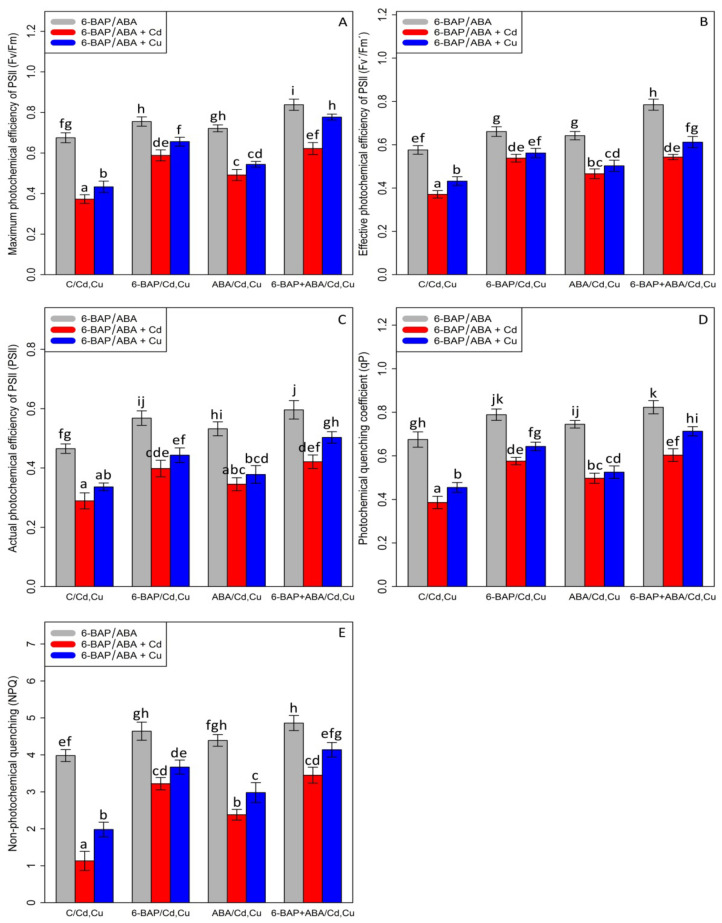
The impact of 6-benzylaminopurine and abscisic acid, alone and in combination, on the maximum photochemical efficiency of PSll (Fv/Fm) (**A**), effective photochemical efficiency of PSll (Fv′/Fm′) (**B**), actual photochemical efficiency of PSll (ɸPSll) (**C**), photochemical quenching coeffi-cient (qP) (**D**), and non-photochemical quenching (NPQ) (**E**) in the one-year-old *Pleioblastus pygmaeus* exposed to 100 µM Cd and 100 µM Cu. In this figure, a, b, c, etc. letters revealed significant differences between 6-BAP, ABA and 6-BAP + ABA in single and in the combined form with 100 µM cadmium and 100 µM copper based on Tukey’s test (*p* < 0.05).

Chlorophyll pigments, including (1) chlorophyll-a (2) chlorophyll b-, and (3) total chlorophyll, as well as (4) carotenoid contents, were measured. Based on the analysis of variance, a significant difference was discovered between the various types of phytohormones in combined and single forms with Cd and Cu (*p* < 0.001). As expected, the concentrations of 100 µM Cd and 100 µM Cu remarkably reduced bamboo chlorophyll pigments and carotenoids with a 47% and 30% reduction in chlorophyll a, a 60% and 39% reduction in chlorophyll b, a 54% and 35% reduction in total chlorophyll, and a 68% and 47% reduction in carotenoids compared with the control treatment. However, 10 µM 6-BAP in combination with Cd and Cu considerably increased chlorophyll pigments. Further, the results indicated that 10 µM ABA had a significant impact on diminishing Cd and Cu toxicity (Figure 2). Nevertheless, 6-BAP was more effective in improving chlorophyll pigments than ABA. According to the results, co-application of 6-BAP + ABA alone and in combination with 100 µM Cd and 100 µM Cu demonstrated the largest increase in chlorophyll pigments by 33%, 72%, and 49% for chlorophyll a, 34%, 121%, and 76% for chlorophyll b, 34%, 94%, and 62% for total chlorophyll, and 50%, 154%, and 110% for carotenoid contents, compared with the control treatments (Figure 2). We proposed that, at optimal concentration (10 µM), these two phytohormones could remarkably increase the photosynthetic process in the plants under heavy metal stress, as seen by an upward trend in chlorophyll fluorescence content in this study. Our results revealed a significant difference between 6-BAP and ABA in both single and combination forms with respect to heavy metal toxicity (Cd and Cu) (*p* < 0.001). However, a similar result of chlorophyll pigments was obtained by chlorophyll fluorescence which showed that the greatest enhancement in chlorophyll fluorescence was attributable to the co-application of 6-BAP and ABA, as evidenced by a 28% rise in actual photochemical efficiency of PSll (ɸPSll), a 21% rise in photochemical quenching coefficient (qP), a 36% rise in effective photochemical efficiency of PSll (Fv′/Fm′), a 24% rise in maximum photochemical efficiency of PSll (Fv/Fm), and a 22% rise in non-photochemical quenching (NPQ), when compared to their control treatment (Figure 3). This enhancement of chlorophyll fluorescence in the treatments involving co-application of 6-BAP + ABA on Cd a Cu was observed in Figure 3. Therefore, we proposed that 6-BAP and ABA, alone or in combination, could significantly increase chlorophyll fluorescence in the plants exposed to Cd and Cu.

### 3.3. The Effect of 6-Benzylaminopurine and Abscisic Acid on the Content of Hydrogen Peroxide (H_2_O_2_), Soluble Proteins (SP), Superoxide Radical (O_2_^•−^), Thiobarbituric Acid Re-Active Substances (TBARS), the Activity of Lipoxygenase (LOX), and Electrolyte Leakage Percentage (EL)

**Figure 4:**
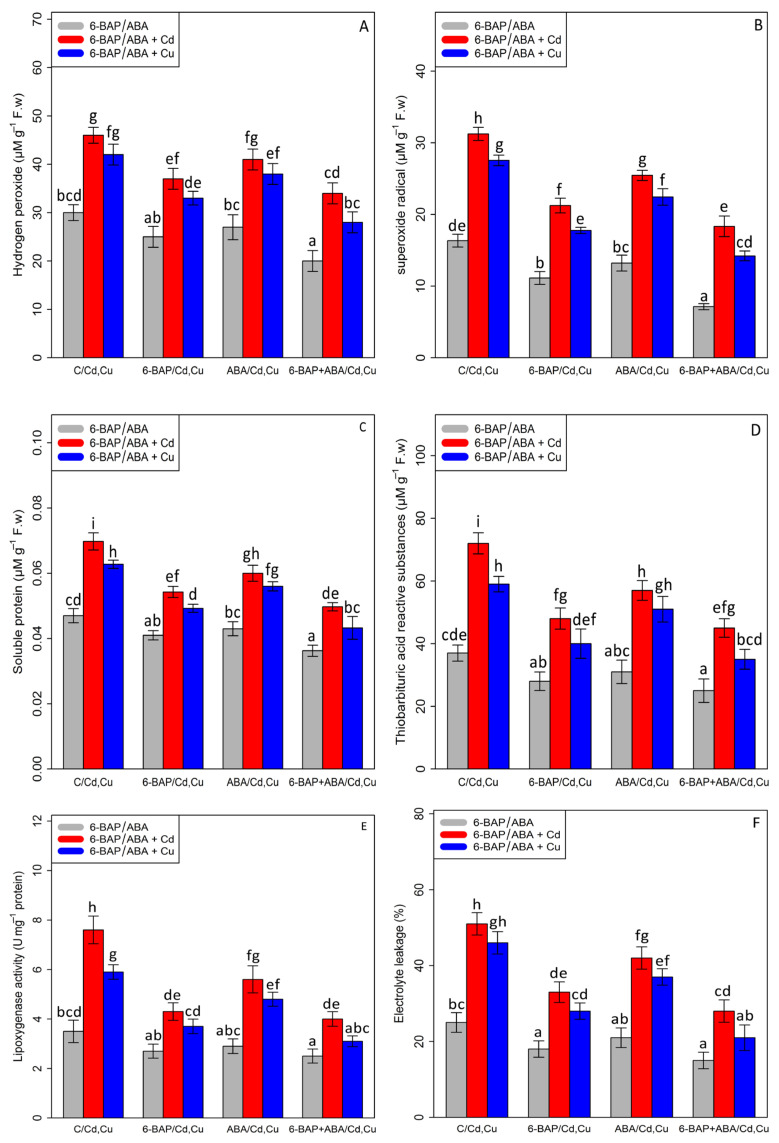
The impact of 6-benzylaminopurine and abscisic acid, alone and in combination, on the content of hydrogen peroxide (H_2_O_2_) (**A**), superoxide radical (O_2_•−) (**B**), soluble proteins (SP) (**C**), thiobarbituric acid reactive substances (TBARS) (**D**), lipoxygenase activity (LOX) (**E**), and electrolyte leakage (EL) (**F**) in *Pleioblastus pygmaeus* exposed to 100 µM Cd and 100 µM Cu. In this figure, a, b, c, etc. letters revealed significant differences between treatments based on Tukey’s test (*p* < 0.05).

Based on the results, a significant difference was found between different treatments of 6-BAP, ABA, and 6-BAP + ABA alone and in combination, with Cd and Cu (*p* < 0.001). In the present study, the results indicated that 100 µM Cd and 100 µM Cu significantly increased the content of hydrogen peroxide (H_2_O_2_—53% and 40%), soluble proteins (SP—46% and 32%), superoxide radical (O_2_^•−^91%, and 68%), thiobarbituric acid reactive substances (TBARS—94% and 59%), the activity of lipoxygenase (LOX—117% and 68%), and electrolyte leakage percentage (EL—104% and 84%) as compared with the control treatment. The addition of 6-BAP significantly decreased ROS compounds and cell damage in the bamboo species under Cd and Cu toxicity by reducing the content of hydrogen peroxide (H_2_O_2_—19% and 21%), soluble proteins (SP—21% and 20%), superoxide radical (O_2_^•−^—31%, and 35%), thiobarbituric acid reactive substances (TBARS—33% and 32%), the activity of lipoxygenase (LOX—43% and 37%), and electrolyte leakage percentage (EL-35% and 39%) as compared with the control treatment. Similar results were revealed by the addition of ABA, which led to a 10% and 9% decrease in the content of H_2_O_2_, a 13% and 9% decrease in the content of SP, a 18% and 18% reduction in the content of (O_2_^•−^), a 20% and 13% reduction in the content of (TBARS), a 26% and 18% decrease in the content of LOX, and a 17% and 19% decrease in the content of EL as compared with their control treatments. In this study, the co-application of 6-BAP+ABA reduced the content of hydrogen peroxide (H_2_O_2_), superoxide radical (O_2_^•−^), thiobarbituric acid reactive substances (TBARS), soluble proteins (SP), the activity of lipoxygenase (LOX), and electrolyte leakage percentage (EL) by 33%, 56%, 32%, 23%, 28%, and 40%, respectively, in comparison to the control treatment (Figure 4). According to data analysis, the co-application of 6-BAP + ABA had the greatest effect on reducing the detrimental impact of ROS compounds and cell damage, which could protect the cell membrane and cell integrity from toxicity. Therefore, the co-application of 6-BAP + ABA in combination with Cd and Cu reduced the negative impact of heavy metals by 26% and caused a 33% reduction in H_2_O_2_ content, a 41% and 48% reduction in O2^• −^ content, a 28% and 30% reduction in SP content, a 37% and 40% reduction in TBARS content, a 47% and 47% reduction in LOX content, and a 45% and 54% reduction in EL content, when compared to their control treatment.

### 3.4. The Effect of the 6-Benzylaminopurine and Abscisic Acid on the Antioxidant Activity in Plants Exposed to Cadmium and Copper

**Figure 5:**
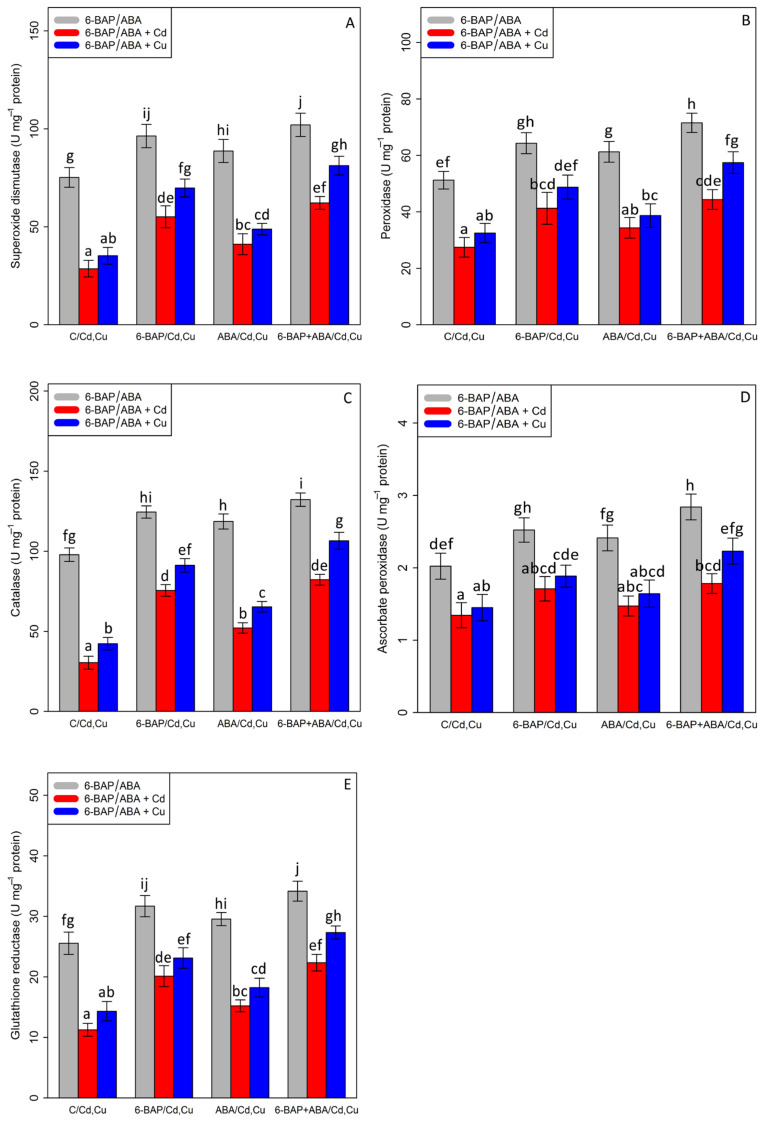
The effect of 6-benzylaminopurine and abscisic acid alone and in combination on the antioxidant enzyme activities; (**A**) superoxide dismutase (SOD), (**B**) peroxidase (POD), (**C**) catalase (CAT), (**D**) ascorbate peroxidase (APX), and (**E**) glutathione reductase (GR) in one-year-old *Pleioblastus pygmaeus* exposed to 100 µM Cd and 100 µM Cu. In this figure, a, b, c, etc. letters revealed significant differences between 6-BAP, ABA and co-application of 6-BAP + ABA in both combined and single form with 100 µM cadmium and 100 µM copper base on Tukey’s test (*p* < 0.05).

The current results demonstrated that there was a significant difference between the two types of phytohormones in their single and combined forms with cadmium and copper (*p* < 0.001). According to Figure 5, antioxidant enzymes activities were significantly reduced in the treatments involving 100 µM Cd and 100 µM Cu. However, it was observed that 10 µM 6-benzyl amino purine individually increased antioxidant enzymes activity in the plants exposed to Cd and Cu with a 92% and 98% increase in SOD, a 78% and 61% increase in GR, a 148% and 115% increase in CAT, a 27% and 29% increase in APX, and a 50% and 50% increase in POD. This enhancement of antioxidants by the addition of ABA in the bamboo species caused a 43% and 38% increase in SOD activity, a 35% and 27% increase in GR activity, a 71% and 54% increase in CAT activity, a 10% and 13% increase in APX, and a 25% and 19% increase in POD activity in the bamboo species under Cd and Cu toxicity as compared with their control treatment. On the other hand, the combination of 6-benzyl amino purine and abscisic acid exhibited the highest value of antioxidant activity, as measured by a 35% enhancement in SOD, a 39% enhancement in POD, a 32% enhancement in CAT, a 40% enhancement in APX, and a 33% enhancement in GR in comparison to the control treatment (Figure 5). This co-application of 6-benzyl amino purine + abscisic acid dramatically improved antioxidant activity in the plant treatments exposed to heavy metal toxicity (Cd and Cu), which demonstrated that the co-application of 6-benzyl amino purine + abscisic acid can be significantly more effective in ameliorating antioxidant activity (Figure 5).

### 3.5. The Effect of 6-Benzyl Amino Purine and Abscisic Acid on the Non-Enzymatic Antioxidant Activity (Flavonols, Tocopherols, and Total Phenolics)

**Figure 6:**
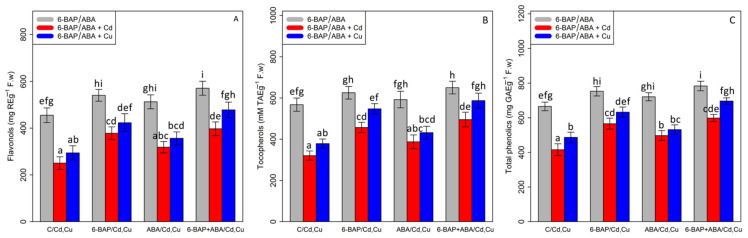
The effect of 6-benzylaminopurine and abscisic acid, single and in combination, on non-antioxidant enzyme activities (Flavonol (**A**), Tocopherol (**B**), and total phenolics (**C**)) in one-year-old *Pleioblastus pygmaeus* expose to 100 µM Cd and 100 µM Cu. In this figure, a, b, c, etc. letters reveal significant differences between control, 6-BAP, ABA and co-application of 6-BAP + ABA in both combined and single form with 100 µM cadmium and 100 µM copper base on Tukey’s test (*p* < 0.05).

According to the results of data analysis for the non-enzymatic antioxidant activity indices (flavonols, tocopherols, and total phenolics), there was a significant difference between various types of phytohormones (6-BAP and ABA) and Cd and Cu (*p* < 0.001). The results showed that the 100 µM Cd and 100 µM Cu significantly reduced non-enzymatic antioxidant activity indices with a 45% and 35% reduction in flavonols, a 43% and 33% reduction in tocopherols, and a 37% and 26% reduction in total phenolics as compared with the control treatment. However, the results of the experiment demonstrated that 6-BAP and ABA, individually and in combination, can increase non-enzymatic antioxidant activity in the plants exposed to heavy metal toxicity where the greatest increases in non-enzymatic antioxidant activity indices under Cd and Cu were related to the bamboo treatments with combination of co-application of 6-BAP + ABA under 100 µM Cd and 100 µM Cu by a 58% and 62% increase in flavanol, a 54% and 55% increase in tocopherol, and a 44% and 43% increase in total phenolics, compared with the control treatments (100 µM Cd and 100 µM Cu) (Figure 6). 

### 3.6. The Effect of 6-Benzyl Amino Purine and Abscisic Acid on the Phenylalanine Ammonia-Lyase and Tyrosine Ammonia-Lyase Activities

**Figure 7:**
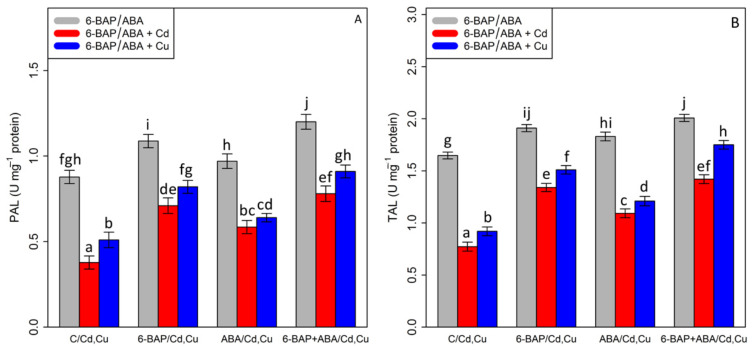
The impact of 6-benzylaminopurine and abscisic acid, alone and in combination, on (**A**) phenylalanine ammonia-lyase (PAL) activity and (**B**) tyrosine ammonia-lyase (TAL) activity in one-year-old *Pleioblastus pygmaeus* exposed to 100 µM Cd and 100 µM Cu. In this figure, a, b, c, etc. letters revealed significant differences between control, 6-BAP, ABA, 6-BAP + ABA, in both combined and single form, with 100 µM cadmium and 100 µM copper based on Tukey’s test (*p* < 0.05).

By analyzing the data, it was shown that there was a substantial difference between 6-BAP and ABA, alone and in combination, with Cd and Cu (*p* < 0.001). According to Figure 7, 100 µM Cd and 100 µM Cu significantly reduced TAL and PAL activity by 53% and 44% in TAL activity and 57% and 41% in PAL activity as compared with the control treatments. On the other hand, the results indicated an increase in TAL and PAL activity with the addition of 6-BAP by 73% and 64% in TAL activity and 91% and 60% in PAL activity (Figure 7). Similar results were obtained by addition of ABA which showed that TAL and PAL activities were increased by 41% and 31% in TAL and 56% and 25% in PAL as compared with their control treatments. However, the greatest increase was attributed to the co-application of 6-BAP and ABA, with a 36% enhancement in PAL activity and a 21% enhancement in TAL activity as compared to their control treatment. The combination of 6-BAP + ABA increased the TAL and PAL activity in the presence of 100 µM Cd and 100 µM Cu, with a 91%, and 56% increase in TAL activity, and 92% and 110% increase in PAL activity as compared with the control treatment.

### 3.7. The Effect of 6-Benzyl Amino Purine and Abscisic Acid on the Content of a Total Thiol, Protein Thiol, and Non-Protein Thiol

By investigating the impact of 6-BAP and ABA on the content of total thiol, protein thiol, and non-protein thiol, a significant difference was observed between the two types of phytohormones when combined with Cd and Cu (*p* < 0.001). The result demonstrated that, with the addition of 100 µM Cd and 100 µM Cu, total thiol, protein thiol, and non-protein were significantly reduced. However, it was found that the addition of 6-BAP, in combination with Cd and Cu greatly raised the thiol concentrations with a 94% and 60% increase in total of thiol, a 48% and 51% increase in protein thiol, and a 76% and 50% increase in non-protein thiol. In the case of ABA, the result showed an increasing trend in thiol state with a 41% and 33% increase in the total thiol, a 27% and 23% increase in protein thiol, and a 49% and 26% in non-protein thiol as compared with their control treatment (Table 4). According to the results, the greatest increase was associated with the co-application of 6-BAP and ABA, with a 33% enhancement in total thiol, a 62% enhancement in protein thiol, and a 17% enhancement in non-protein content when compared to their control treatment. Therefore, the results demonstrated that the co-application of 6-BAP and ABA exhibited the greatest increase in the treatments under Cd and Cu. Compared to their respective control treatments, there was a 99% and 81% increase in the total thiol, a 64% and 82% increase in protein thiol, and a 85% and 67% increase in non-protein thiol under Cd and Cu toxicity.

### 3.8. The Effect of 6-Benzyl Amino Purine and Abscisic Acid on the Proline (Pro) Contents, Glycine Betaine (GB), and Glutathione (GSH)

Proline (Pro), glycine betaine (GB), and glutathione (GSH) content can play a significant role in protecting cells from oxidative stress and preserving redox homeostasis. There was a significant difference between the two types of phytohormone, alone and in combination, with 100 µM Cd and 100 µM Cu (*p* < 0.001) (Table 5). According to the results, the contents of proline (Pro), glycine betaine (GB), and glutathione (GSH) significantly increased with the addition of 6-BAP and ABA. However, the 6-BAP showed a more significant impact on increasing proline (Pro), glycine betaine (GB), and glutathione (GSH) content. Additionally, it was found that the greatest increase in the treatments under Cd and Cu was associated with the co-application of 6-BAP + ABA in combination with 100 µM Cd and 100 µM Cu, with a 136% and 117% rise in proline content, a 54% and 54% rise in glycine betaine content, and a 97% and 109% rise in glutathione content, when compared to the control treatment. In contrast, our results demonstrated that the treatments of 100 µM Cd and 100 µM Cu significantly reduced the content of proline (Pro), glycine betaine (GB), and glutathione (GSH) with a 60% and 48% reduction in proline content, a 36% and 29% reduction in glycine betaine, and a 55% and 47% reduction in glutathione content in comparison to the control treatments.

### 3.9. The Effect of 6-Benzyl Amino Purine and Abscisic Acid on the Accumulation of Cadmium and Copper in Stems, Roots, and Shoots in Species of Bamboo

Decreased metal accumulation in plant organs can be one of the major mechanisms for enhancing a plant’s tolerance to metal toxicity. In the course of the reduction of accumulation of heavy metals in leaves, stems, and shoots, our results revealed a significant difference between different types of phytohormones, in single and combination form with Cd and Cu (*p* <0.001) (Table 6). The result showed that treatments with 100 µM Cd and 100 µM Cu had maximum cadmium and copper accumulation in the plant organs (leaves, stem, and root), which showed the negative impact of the metals’ toxicity on plant growth. Additionally, the results indicated that the addition of 6-BAP and ABA could significantly reduce Cd and Cu accumulation in leaves, stem, and shoot. Therefore, the co-application of 6-BAP and ABA resulted in the greatest reduction of Cd and Cu in leaves, stem, and root, i.e., a 35% and 45% decrement in leaves, a 36% and 44% decrement in stem, and a 34% and 37% decrement in root, when compared to their respective control treatments. In addition, the results demonstrated that 10 µM 6-BAP and 10 µM ABA in the single form had the ability to reduce Cd and Cu content in the plant organs by 25%, 37%, with an 11%, 16% decrease in leaves, 31%, 36%, and 17%,18% decrease in stem, as well as 29%, 32% and 16%, 13% decrease in plant roots respectively relative to their control treatment (Table 6).

### 3.10. The Effect of 6-Benzyl Amino Purine and Abscisic Acid on the Bioaccumulation Factor (BAF), Tolerance Index (TI), and Translocation Factor (TF)

Bioaccumulation factor (BAF), tolerance index (TI), and translocation factor (TF) were calculated for the purpose of determining plant tolerance to heavy metal stress. Our findings indicated that there was a considerable difference between the two types of phytohormones (6-BAP and ABA) in single and combined forms under Cd and Cu in bioaccumulation factor (BAF) (*p* < 0.001). As expected, the addition of 6-BAP and ABA reduced BAF in leaves. However, the greatest impact on reduction of BAF occurred with the co-application of 10 µM 6-BAP + 10 µM ABA. In the single forms, 10 µM 6-BAP had the lowest BAF in shoots which can be attributed to the decreased translocation of Cd and Cu from root to stem and shoot. In this regard, the results showed a significant reduction in translocation factor between bamboo treatments (TF) (*p* < 0.001). Therefore, the results demonstrated that 10 µM 6-BAP, 10 µM ABA and 10 µM 6-BAP + 10 µM ABA considerably reduced the translocation factor in leaves, leading to an increase in the plant tolerance index in shoots and roots. A similar significant difference was observed for tolerance indices in the root and shoot in our bamboo species. Therefore, we suggest that the addition of 6-BAP + ABA had the greatest impact on improving the shoot and root bamboo tolerance under Cd and Cu, which significantly increased plant resistance to toxicity, which was attained by lowering the translocation factor and bioaccumulation factor in stem and leaves (Table 7).

## 4. Discussion

In this research, bamboo species treated with 6-BAP and ABA had a strong ability to increase root fresh weight/dry weight, shoot fresh weight/dry weight, and shoot length in the presence of Cd and Cu toxicity. Numerous studies have demonstrated that growth modulator (PGMs) factors in plants, i.e., 6-BAP and ABA, enhance plant growth under different abiotic stresses [23,62,63]. By the addition of 6-BAP and ABA under Cd and Cu, plant growth and development, as well as plant biomass, increase for a number of reasons, including reduced cell membrane damage [25], increased transpiration rate, enhanced photosynthetic activity, and amino acids content [24,64]. Enhancing the performance of chlorophyll is an indicator of the photosynthesis efficiency of plants [65]. The accumulation of ROS can reduce chloroplastic thylakoid and grana in the plant cells [66], as well as impair CO_2_ assimilation and lower carbon-capture in plants [67]. On the other hand, it has been observed that 6-BAP enhances photosynthetic indices in *B. juncea* when exposed to cadmium [13]. In addition, the same result has been reported for any levels of ABA in enhancing chlorophyll pigments in *B. campestris* under Cd stress [68]. In plants exposed to Cd and Cu, 100 µM Cd and 100 µM Cu dramatically increased photosynthetic pigments and chlorophyll fluorescence. This is consistent with other studies [14,69,70,71]. In one study on tomatoes, 6-BAP was found to boost chlorophyll formation by reducing heavy metals toxicity, which ultimately led to an increase in tomato cell division and growth [72,73]. In addition, abscisic acid, a pivotal signaling molecule synthesized by chloroplast [74], increases the tolerance of tomatoes against cobalt (Co) toxicity [14]. All these studies were in line with our results on bamboo species, which demonstrated the 6-BAP and ABA greatly boosted photosynthetic pigments and chlorophyll fluorescence under Cd and Cu, leading to the increased plant growth. The accumulation of ROS in plant cells exposed to excessive levels of heavy metals can have a detrimental effect on macromolecules and cyto-membranes, disrupting numerous plant physiology processes [75,76]. In the current research work, our results demonstrated that ROS compounds accumulate and increase in plants grown in Cd and Cu excess environments. This could accelerate plant oxidation and increase plant membrane damage and lipoperoxidation [77,78]. This is while the addition of 6-BAP and ABA reduced the content of H_2_O_2_ and O_2_ in the bamboo plant, which could protect the plant cell membrane by reducing lipid peroxidation and electrolyte leakage. Hence, our results demonstrated that 6-BAP and ABA, both alone and in combination, significantly reduce H_2_O_2_, O_2_^•−^, TBARS, EL, SP, and LOX in the plants exposed to 100 µM Cd and 100 µM Cu. Numerous researchers have demonstrated the impact of 6-BAP and ABA on lipoperoxidation and ROS [69,70,71,72,73,74,75,76,77,78,79]. One of the strategies employed by plants under heavy metal stress is to increase their cellular soluble protein, which can help to decrease the intracellular osmotic potential and provide the plants with normal water status as well as preserving the physiology process in the cell. Therefore, the enhanced protein content can be one of the indicators of heavy metal toxicity in plants [80,81]. According to our results, the content of soluble protein in the bamboo species under Cd and Cu toxicity increased. However, the addition of phytohormones reduced soluble protein content in the metal-stressed bamboo, from which it can be concluded that the addition of 6-BAP and ABA ameliorated metal toxicity in the bamboo species. The combination of 6-BAP and ABA stimulated antioxidant and non-antioxidant activity, resulting in a decrease in ROS compounds, improvement of membrane peroxidation, and maintenance of cell integrity. This demonstrated that 10 µM 6-BAP and 10 µM ABA, in both individual and combination forms, increased antioxidant activity, including SOD, GR, CAT, APX, and POD. SOD and APX are two essential antioxidants known as the frontline of a plant’s defense mechanism against ROS compounds. CAT, POD, and GR are oxidative stress biomarkers that play a significant role in diminishing plant oxidative stress caused by ROS compounds [75]. In recent years, numerous researchers have demonstrated that 6-BAP and ABA enhance the plant’s antioxidant activity under diverse abiotic stresses and different heavy metals. This results in an increase in ROS scavenging, membrane stabilization, and a reduction in oxidative stress [20,21,62,63,64,65,66,67,68,69]. According to Kamran et al. [82], the co-application of 6-BAP + ABA increased antioxidant activity (APX, POD, SOD, and CAT) and lessened oxidative damage in tomatoes exposed to cobalt, which is consistent with the results of the present study. The activation of gene expression associated with the activity of antioxidant enzymes and non-enzyme antioxidants is mediated by ABA signals. Recent research on grapevine (*Vitis vinifera* L.) discovered that ZEP gene expression is involved in ABA biosynthesis, and that ABA levels in leaves are higher than in other plant parts [83,84]. This appears to be one reason why the addition of ABA increased antioxidant activity in this study. 6-BAP, as a member of the cytokine group, in stressed wheat leaves has been demonstrated to reduce ROS compounds by decreasing ROS at the cellular level [83]. However, the majority of cytokine (CK) group efficiency is related to alteration in CK levels. It has been reported that lowering CK levels increases plant performance under stress conditions, which directly contributes to the increase in ABA concentration under stress conditions. Thus, ABA can regulate the CK concentration in plants under stress via mechanisms such as the activation of special enzymes involved in CK degradation or gene expression in response to the control of CK biosynthesis [85].

PAL and TAL are substantial biosynthetic enzymes, along with phenolic compounds [86]. Despite the fact that 100 µM Cd and 100 µM Cu reduced TAL and PAL activity, the addition of 6-BAP and ABA counteracts these effects by increasing the activity of PAL and TAL in the plants exposed to Cd and Cu, which demonstrates the involvement of these two phytohormones, in boosting phenolic content under HMs. Phenolic compounds are non-enzymatic antioxidants that inhibit reactive oxygen species scavenging [86]. On the other hand, ABA accelerated phenolic compounds biosynthesis, which could accumulate in plant leaves [87]. In one study, ABA elevated the synthesis of phenolic acids and the activity of PAL and TAT in the hairy root of *S. miltiorrhiza* [88]. According to our findings, 10 µM 6-BAP and 10 µM ABA, alone or in combination, significantly increased phenolic content, including total phenolic, flavonols, and tocopherol, in the bamboo species exposed to Cd and Cu. This suggests that 6-BAP and ABA enhanced plant defense mechanisms against ROS compounds by improving secondary metabolisms, such as the stimulation of non-enzyme activity and the increase in the generation of active plants ingredients. Numerous studies have demonstrated the necessity of 6-BAP and ABA for plants to increase and maintain their phenolic content [69,89,90]. 

Thiols (-SH) are essential due to their varied functions in the plant biological system. They consist of two types of protein thiols (PSH) as well as non-protein thiols (NPSH) that can influence several physiological and pathological functions of plants [91]. There have been reports that certain thiol-containing molecules can form complexes with heavy metals, which can be used as antidotes for heavy metal toxicity [1]. As thiol-mediated compounds, phytohormones function as regulatory factors for the reduction of heavy metal stress [92]. While 100 µM Cd and 100 µM Cu reduced protein thiols (PSH) and non-protein thiols (NPSH), the addition of two phytohormones (6-BAP and ABA), individually and in combination, increased protein thiols, non-protein thiols, and total thiols in the plants exposed to cadmium and copper. 

Proline increases plant tolerance by scavenging ROS compounds and preventing macromolecule dehydration under stressful conditions, as measured by osmoprotectants and antioxidants, respectively [93]. In plants under stress, proline increases antioxidant activity [94]. This was evident throughout this study. Under Cd and Cu, 6-BAP and ABA conspicuously enhanced proline content, which may be related to the role of ABA in stimulating proline synthesis from glutamic acid [95]. Glutathione (GSH) is a vital indicator in the amelioration of plant stress, playing a crucial role in the regulation of redox balance as a buffering system. This can be found in every plant cell organelle [96]. Despite the fact that 10 µM ABA and 10 µM 6-BAP reduced the GSH levels, the addition of 6-BAP and ABA increased the GSH content. Numerous studies have reported that proline and GSH can prevent metal toxicity in plants [97,98]. Glycine betaine (GB), as a compatible substance, can regulate plant osmotic conditions under stress [86]. On the other hand, it has been reported that the accumulation of glycine betaine in plants can increase the accumulation of photosynthetic pigments in chloroplasts, hence enhancing the protection efficacy of photosystem II (PSII) [65]. This can ultimately ameliorate plant photosynthesis indexes under stressful conditions. 

Cd and Cu accumulation increased in this study under 100 µM Cd and 100 µM Cu treatments. However, the addition of 6-BAP and ABA, alone or in combination, reduced cadmium and copper accumulation in stem, leaves, and roots. The potential of phytohormones to diminish metal translocation (TF) from root to aerial portions was demonstrated by the fact that metal accumulation in plant roots was greater than in leaves and stems. In fact, the result demonstrates that plant roots are more susceptible to metal toxicity than plant shoots, therefore small heavy metals can transfer to the stem and leaves [77,78]. Consequently, our results indicated that ABA and 6-BAP considerably limit the TF from the root to the stem and leaves. It has been observed that ABA can inhibit heavy metal translocation from the root to aerial parts, resulting in an accumulation of metal ions on the root surface [99] and an increase in the phytoremediation potential of plants in contaminated areas. This has been addressed in some research on *P. euphratica* and *B. campestris* [68,100]. Additionally, tomato [82] and *Solanum melongena* [71] have provided some evidence that 6-BAP has the capacity to inhibit the accumulation of heavy metals in plant organs. These are in agreement with our findings regarding the bamboo species. According to Table 7 of the current study, the combination of 6-BAP and ABA considerably lowers the metal TF from the root to the stem and shoot. This reduces metal bioaccumulation and bioaccumulation factors in the aerial plant organs, resulting in a higher plant tolerance index for the bamboo in the presence of Cd and Cu toxicity. However, the results demonstrated that 6-BAP and ABA have a positive impact on the metal accumulation on the root surface, demonstrating that the plant growth modulators (PGMs) factors, i.e., 6-BAP and ABA, can increase the bamboo phytoremediation in the polluted soils and lead to the elimination of metal toxicity in the surrounding environment.

It is reported that the mechanism involved in the reduction of metal toxicity by 6-ABP and ABA might be related to the role PGMs (ABA and 6-BAP), which triggers the production of different types of chelators such as phytochelatins (PCs), metallothionein, and decreases glutathione (GSH) content to diminish the toxicity and mobility of ions in binding sites [101]. Besides, it accumulates amino acids (methionine and γ-aminobutyrate) [64] and boosts the transpiration and photosynthesis rate [14]. However, the underlying mechanism of ABA and 6-BAP efficiency is not fully understood, and it varies between species and depends on the type and concentration of heavy metals. Our results indicated that the applications of 6-BAP and ABA may reduce heavy metal toxicity in the bamboo plants through a number of mechanisms, including an enhancement of antioxidant and non-antioxidant activity, thiol protein, proline, and glutathione contents, as well as a reduction in metal translocation and metal accumulation in leaves and stems. This can finally safeguard plant cell membranes and cell integrity as well as boost plant tolerance to Cd and Cu toxicity.

## 5. Conclusions

According to our results, plant growth modulators (PGMs), such as 6-BAP and ABA, alone or in combination, ameliorated oxidative stress in the bamboo plant under cadmium and copper stress. This could protect cell membranes from lipoperoxidation and reduce electrolyte leakage by stimulating antioxidant and non-antioxidant activities, increasing PAL and TAL activities, boosting proline, glutathione, and glycine betaine contents, as well as accumulating protein thiols (PSH) and non-protein thiols (NPSH). This could potentially improve chlorophyll pigments and chlorophyll fluorescence and promote plant growth under toxicity. We suggested that 6-BAP and ABA, alone or in combination, increase plant resistance to cadmium and copper toxicity via several key mechanisms, including (1) the activation of the plant defense mechanism and the secondary metabolism process, (2) inhibition of metal transfer from the root to the stem and shoot, (3) reduction of metal accumulation, and (4) enhancement of plant photosynthesis. We concluded that the co-application of ABA and 6-BAP in combination form is more effective in increasing bamboo plant tolerance to Cd and Cu. We also suggested that 6-BAP and ABA can improve the bamboo phytoremediation potential in the polluted areas. These results can improve our knowledge in order to utilize the potential of phytohormones in boosting bamboo plant tolerance, which can contribute to the proper selection of different species of bamboo for phytoremediation purposes. This is a preliminary research and more work needs to be done with different bamboo species in this area to identify the optimum levels of various phytohormones in single and combination forms. Moreover, understanding the mechanisms involved in conferring such tolerance is one of the priorities of our future work.

## Figures and Tables

**Table 1:** The experimental design.

Treatments	Concentrations
Control	0
Cd	100 µM Cd
Cu	100 µM Cu
6-BAP	10 µM 6-BAP
6-BAP + Cd	10 µM 6-BAP + 100 µM Cd
6-BAP + Cu	10 µM 6-BAP + 100 µM Cu
ABA	10 µM ABA
ABA + Cd	10 µM ABA + 100 µM Cd
ABA + Cu	10 µM ABA + 100 µM Cu
ABA + 6-BAP	10 µM 6-BAP + 10 µM ABA
ABA + 6-BAP + Cd	10 µM 6-BAP + 10 µM ABA + 100 µM Cd
ABA + 6-BAP + Cu	10 µM 6-BAP + 10 µM ABA + 100 µM Cu

**Table 2:** The impact of 6-benzylaminopurine and abscisic acid, alone and in combination, on root fresh weight, root dry weight, shoot fresh weight, shoot dry weight, FW/DW in root, FW/DW in shoot and shoot length in the one-year-old *Pleioblastus pygmaeus* exposed to 100 µM Cd and 100 µM Cu. In this table, a–j letters revealed significant differences in the treatments of each index based on Tukey’s test (*p* < 0.05).

Treatments	FW (g)		DW (g)		FW/DW Root	FW/DW Shoot	Shoot Length (cm)
	Root	Shoot	Root	Shoot			
C	15.55 ± 0.46 ^bcde^	10.32 ± 0.38 ^efg^	1.13 ± 0.04 ^fg^	0.85 ± 0.01 ^gh^	13.37 ± 1.37 ^ab^	12.11 ± 0.12 ^ab^	14.45 ± 0.44 ^def^
Cd	14.02 ± 0.45 ^a^	5.75 ± 0.43 ^a^	0.74 ± 0.02 ^a^	0.47 ± 0.03 ^a^	18.86 ± 0.16 ^f^	12.08 ± 0.10 ^ab^	10.88 ± 0.69 ^a^
Cu	14.55 ± 0.42 ^ab^	6.64 ± 0.47 ^ab^	0.87 ± 0.02 ^b^	0.55 ± 0.03 ^b^	16.70 ± 0.26 ^e^	12.03 ± 0.13 ^ab^	12.02 ± 0.44 ^ab^
6-BAP	16.19 ± 0.32 ^de^	11.39 ± 0.45 ^gh^	1.26 ± 0.03 ^ij^	0.93 ± 0.02 ^ij^	12.80 ± 0.44 ^a^	12.19 ± 0.01 ^bc^	15.54 ± 0.40 ^f^
6-BAP + Cd	15.02 ± 0.40 ^abcd^	8.78 ± 0.63 ^cd^	1.02 ± 0.03 ^de^	0.68 ± 0.03 ^de^	14.74 ± 0.25 ^cd^	12.76 ± 0.18 ^f^	13.46 ± 0.47 ^cd^
6-BAP + Cu	15.23 ± 0.43 ^abcd^	9.97 ± 0.61 ^def^	1.10 ± 0.03 ^f^	0.80 ± 0.02 ^fg^	13.83 ± 0.16 ^abc^	12.23 ± 0.07 ^bc^	14.21 ± 0.40 ^de^
ABA	15.80 ± 0.59 ^cde^	11.16 ± 0.63 ^fgh^	1.21 ± 0.02 ^hi^	0.90 ± 0.01 ^hij^	13.03 ± 0.76 ^ab^	12.35 ± 0.05 ^cd^	14.89 ± 0.44 ^ef^
ABA + Cd	14.67 ± 0.53 ^abc^	7.12 ± 0.40 ^b^	0.93 ± 0.02 ^bc^	0.56 ± 0.01 ^bc^	15.75 ± 0.25 ^de^	12.54 ± 0.09 ^def^	12.55 ± 0.49 ^bc^
ABA + Cu	14.89 ± 0.52 ^abc^	7.56 ± 0.46 ^bc^	0.98 ± 0.01 ^cd^	0.63 ± 0.01 ^cd^	15.14 ± 0.50 ^cd^	11.95 ± 0.05 ^a^	13.34 ± 0.43 ^cd^
6-BAP + ABA	16.72 ± 0.53 ^e^	11.95 ± 0.51 ^h^	1.29 ± 0.02 ^j^	0.96 ± 0.03 ^j^	12.92 ± 0.33 ^ab^	12.37 ± 0.02 ^cde^	17.34 ± 0.35 ^g^
6-BAP + ABA + Cd	15.14 ± 0.57 ^abcd^	9.36 ± 0.52 ^de^	1.06 ± 0.02 ^ef^	0.74 ± 0.02 ^ef^	14.19 ± 0.25 ^bc^	12.59 ± 0.04 ^ef^	13.97 ± 0.47 ^de^
6-BAP + ABA + Cu	15.77 ± 0.57 ^bcde^	10.65 ± 0.36 ^fg^	1.18 ± 0.01 ^gh^	0.88 ± 0.02 ^hi^	13.28± 0.36 ^ab^	12.04 ± 0.04 ^ab^	14.86 ± 0.39 ^ef^

**Table 3:** The increasing and decreasing percentage of fresh/dry weight of root and shoot as well as shoot length in the bamboo species exposed to 100 µM cadmium and 100 µM copper in single and combined forms with 10 µM 6-BAP and 10 µM ABA, compared to the control treatment. ↑ indicates increases and ↓ indicates decreases.

Treatments	FW Root	FW Shoot	DW Root	DW Shoot	Shoot Length
Cd	10% ↓	44% ↓	34% ↓	44% ↓	24% ↓
Cu	6% ↓	35% ↓	22% ↓	35% ↓	16% ↓
6-BAP	4% ↑	10% ↑	11% ↑	9% ↑	7% ↑
6-BAP + Cd	3% ↓	15% ↓	9% ↓	19% ↓	6% ↓
6-BAP + Cu	2% ↓	3% ↓	2% ↓	5% ↓	1% ↓
ABA	2% ↑	8% ↑	7% ↑	6% ↑	3% ↑
ABA + Cd	5% ↓	31% ↓	17% ↓	33% ↓	13% ↓
ABA + Cu	4% ↓	26% ↓	13% ↓	25% ↓	7% ↓
6-BAP + ABA	7% ↑	16% ↑	14% ↑	13% ↑	20% ↑
6-BAP + ABA + Cd	2% ↓	9% ↓	6% ↓	12% ↓	3% ↓
6-BAP + ABA + Cu	1% ↑	3% ↑	5% ↑	4% ↑	3% ↑

**Table 4:** The impact of 6-benzylaminopurine and abscisic acid, alone and in combination, on total thiol, protein thiol, and non-protein thiol in the one-year-old *Pleioblastus pygmaeus*, exposed to 100 µM Cd as well as 100 µM Cu. In this table, a, b, c, etc. letters revealed significant differences between control, 6-BAP, ABA, and co-application of 6-BAP + ABA in both combined and single forms with 100 µM cadmium and 100 µM copper based on Turkey’s test (*p* < 0.05).

Treatments	Total Thiol μmol ^−1^ g FW	Protein Thiol μmol g ^−1^ FW	Non-Protein μmol g ^−1^ FW
Control	20.45 ± 0.67 ^f^	8.14 ± 0.49 ^ef^	14.0 ± 0.76 ^fgh^
Cd	8.55 ± 0.55 ^a^	4.34 ± 0.48 ^a^	6.7 ± 0.77 ^a^
Cu	11.66 ± 0.69 ^b^	4.98 ± 0.51 ^ab^	8.8 ± 0.73 ^b^
6-BAP	25.34 ± 0.86 ^h^	10.75 ± 0.46 ^h^	15.8 ± 0.57 ^hi^
6-BAP + Cd	16.66 ± 0.79 ^cd^	6.44 ± 0.46 ^cd^	11.8 ± 0.98 ^cde^
6-BAP + Cu	18.77 ± 0.63 ^e^	7.56 ± 7.56 ^e^	13.2 ± 0.45 ^efg^
ABA	23.55 ± 0.46 ^g^	10.04 ± 0.49 ^gh^	15.3 ± 0.83 ^hi^
ABA + Cd	12.11 ± 0.63 ^b^	5.55 ± 0.41 ^bc^	10.0 ± 0.70 ^bc^
ABA + Cu	15.55 ± 0.51 ^c^	6.12 ± 0.41 ^cd^	11.1 ± 0.66 ^cd^
6-BAP + ABA	27.22 ± 0.56 ^i^	13.25 ± 0.36 ^i^	16.4 ± 0.60 ^i^
6-BAP + ABA + Cd	17.11 ± 0.57 ^d^	7.13 ± 0.46 ^de^	12.4 ± 0.62 ^def^
6-BAP + ABA + Cu	21.11 ± 0.34 ^f^	9.11 ± 0.36 ^fg^	14.7 ± 0.87 ^ghi^

**Table 5:** The impact of 6-benzylaminopurine and abscisic acid, alone and in combination, on proline (Pro), glycine betaine (GB), and glutathione (GSH) content in the one-year-old *Pleioblastus pygmaeus* exposed to 100 µM Cd and 100 µM Cu. In this table, a, b, c, etc. letters revealed significant differences between the treatments according to Tukey’s test (*p* < 0.05).

Treatments	Proline (Pro) µg g^−1^ FW	Glycine Betaine (GB) µg g^−1^ FW	Glutathione (GSH) µmol g^−1^ FW
Control	492.11 ± 39.36 ^efg^	803.25 ± 36.95 ^e^	97.66 ± 4.90 ^fg^
Cd	194.35 ± 45.01 ^a^	507.50 ± 19.01 ^a^	43.34 ± 4.33 ^a^
Cu	254.44 ± 37.99 ^ab^	564.75 ± 40.42 ^ab^	51.36 ± 5.00 ^ab^
6-BAP	560.58 ± 44.28 ^gh^	905.50 ± 38.49 ^fg^	121.33 ± 4.50 ^ij^
6-BAP + Cd	387.70 ± 44.59 ^cd^	703.25 ± 39.19 ^cd^	78.44 ± 4.19 ^cd^
6-BAP + Cu	464.43 ± 44.96 ^def^	787.00 ± 41.28 ^de^	92.33 ± 4.62 ^ef^
ABA	542.21 ± 32.74 ^efgh^	874.75 ± 42.22 ^ef^	115.44 ± 4.92 ^hi^
ABA + Cd	306.66 ± 31.81 ^bc^	601.00 ± 36.00 ^b^	62.11 ± 3.40 ^b^
ABA + Cu	355.77 ± 36.27 ^c^	650.00 ± 35.72 ^bc^	74.33 ± 4.49 ^c^
6-BAP + ABA	620.50 ± 34.01 ^h^	972.50 ± 38.75 ^g^	128.55 ± 3.38 ^j^
6-BAP + ABA+ Cd	458.87 ± 30.24 ^de^	786.00 ± 40.62 ^de^	85.44 ± 4.90 ^de^
6-BAP + ABA+ Cu	553.43 ± 29.81 ^fgh^	872.00 ± 38.41 ^ef^	107.44 ± 4.38 ^g^

**Table 6:** The impact of 6-benzylaminopurine and abscisic acid, alone and in combination, on accumulation of cadmium and copper in the plant organs (leaves, stem and root) exposed to 100 µM Cd and 100 µM Cu. In this table, a, b, c, etc. letters revealed significant differences between control, 6-BAP, ABA, and the co-application of 6-BAP + ABA in both combined and single forms with 100 µM cadmium and 100 µM copper based on Tukey’s test (*p* < 0.05).

Treatments	Heavy Metals Content	Heavy Metal Accumulation (Leaves)	Heavy Metal Accumulation (Stem)	Heavy Metal Accumulation (Root)
µmol L^−1^	µmol L^−1^	µmol L^−1^	µmol L^−1^	µmol L^−1^
C	0	0	0	0
Cd	100µM Cd	25.22 ± 0.35 ^g^	29.65 ± 0.54 ^i^	37.55 ± 0.83 ^i^
Cu	100µM Cu	22.87 ± 0.52 ^f^	26.33 ± 0.51 ^h^	33.22 ± 0.70 ^h^
6-BAP	0	0	0	0
6-BAP + Cd	100µM Cd	18.75 ± 0.43 ^e^	20.21 ± 0.47 ^e^	26.55 ± 0.91 ^e^
6-BAP + Cu	100 µM Cu	14.33 ± 0.52 ^c^	16.66 ± 0.61 ^c^	22.33 ± 0.83 ^c^
ABA	0	0	0	0
ABA + Cd	100 µM Cd	22.44 ± 0.51 ^f^	24.56 ± 0.52 ^g^	31.22 ± 0.85 ^g^
ABA + Cu	100µM Cu	19.22 ± 0.58 ^e^	21.44 ± 0.51 ^f^	28.77 ± 0.77 ^f^
6-BAP + ABA	0	0	0	0
6-BAP + ABA + Cd	100 µM Cd	16.23 ± 0.25 ^d^	18.76 ± 0.63 ^d^	24.55 ± 0.66 ^d^
6-BAP + ABA + Cu	100 µM Cu	12.55 ± 0.27 ^b^	14.66 ± 0.39 ^b^	20.66 ± 0.76 ^b^

**Table 7:** Changes in the tolerance index of shoots and roots, translocation factor, and bioaccumulation factor in response to 10 µM 6-BAP + 10 µM ABA in combined or single forms when exposed to 100 µM cadmium and 100 µM copper, as compared to the control treatment. Each data point is the mean ± standard error for four repetitions. In this table, a, b, c, etc. letters revealed significant differences between treatments based on Tukey’s test (*p* < 0.05).

Treatments	Translocation Factor (TF) (Leaves)	Tolerance Index (TI) (Shoot)	Tolerance Index (TI) (Root)	Bioaccumulation Factor (Leaves) (BF)
0	0.00 ± 0.00 ^c^	1.00 ± 0.00 ^fg^	1.00 ± 0.00 ^fgh^	0.00 ± 0.00 ^g^
Cd	0.71 ± 0.023 ^ab^	0.55 ± 0.05 ^a^	0.65 ± 0.01 ^a^	0.25 ± 0.003 ^f^
Cu	0.70 ± 0.03 ^b^	0.65 ± 0.05 ^ab^	0.77 ± 0.02 ^b^	0.22 ± 0.005 ^e^
6-BAP	0.00 ± 0.00 ^c^	1.09 ± 0.05 ^gh^	1.12 ± 0.07 ^ij^	0.00 ± 0.00 ^g^
6-BAP + Cd	0.66 ± 0.03 ^ab^	0.80 ± 0.04 ^cd^	0.90 ± 0.01 ^cde^	0.18 ± 0.004 ^d^
6-BAP + Cu	0.64 ± 0.04 ^a^	0.94 ± 0.04 ^ef^	0.97 ± 0.01 ^efg^	0.14 ± 0.005 ^b^
ABA	0.00 ± 0.00 ^c^	1.06 ± 0.03 ^fgh^	1.07 ± 0.06 ^hig^	0.00 ± 0.00 ^g^
ABA + Cd	0.68 ± 0.01 ^ab^	0.66 ± 0.03 ^ab^	0.82 ± 0.01 ^bc^	0.22 ± 0.005 ^e^
ABA + Cu	0.67 ± 0.02 ^a^	0.74 ± 0.03 ^bc^	0.86 ± 0.02 ^cd^	0.19 ± 0.005 ^d^
6-BAP + ABA	0.00 ± 0.00 ^c^	1.13 ± 0.06 ^h^	1.14 ± 0.04 ^j^	0.00 ± 0.00 ^g^
6-BAP + ABA + Cd	0.66 ± 0.01 ^ab^	0.87 ± 0.05 ^de^	0.94 ± 0.02 ^def^	0.16 ± 0.002 ^c^
6-BAP + ABA + Cu	0.60 ± 0.01 ^a^	1.03 ± 0.05 ^fgh^	1.05 ± 0.03 ^ghi^	0.12 ± 0.002 ^a^

## Data Availability

The data presented in this study are available in article.

## Abbreviations

6-BAP	Benzylaminopurine
ABA	Abscisic acid
Cd	Cadmium
Cu	Copper
SOD	Superoxide dismutase
GR	Glutathione reductase
CAT	Catalase activity
APX	Ascorbate peroxidase
POD	Peroxidase
TAL	Tyrosine ammonia-lyase
PAL	Phenylalanine ammonia-lyase
GB	Glycine betaine
GSH	Glutathione
Pro	Proline
ICP	Inductively coupled plasma
TF	Translocation Factor
Ti	Tolerance Index
BAF	Bio Accumulation Factor
DW	Dry Weight
FW	Fresh Weight
(NPQ),	Non-Photochemical Quenching
(qP)	Photochemical quenching coefficient
(ɸPSll);	Actual photochemical efficiency of PSll
(Fv′/Fm′),	Effective photochemical efficiency of PSll
(Fv/Fm);	Maximum photochemical efficiency of PSll
(TBARS),	Thiobarbituric acid reactive substances
SP	Soluble Protein
EL	Electrolyte Leakage
LOX	Lipoxygenase activity
H_2_O_2_	Hydrogen peroxide
O_2_^•−^	Superoxide radical
ROS	Reactive oxygen species
MDA	Malondialdehyde
NTFP	Non-timber forest product
KT	Kinetin
IAA	Indole-3-acetic acid
LN	Liquid nitrogen
CFI	Fluorescence imager machine (CFI)
(NO2)	Nitrogen dioxide radical
NBT	Nitro blue tetrazolium
TAC	Tocopherol Acetate Standard
TTs	Total Thiol
NPTs, NPSH	Non-Protein Thiols
PT, PSH	Protein Thiol
AAS	Atomic Absorption Spectrometry
CRD	Completely Randomized Design
PGMs	Plant Growth Modulators
PCs	Phytochelatins
